# Familial chronic lymphocytic leukemia in two siblings with ATM/13q14 deletion and a similar pattern of clonal evolution

**DOI:** 10.1038/bcj.2015.50

**Published:** 2015-07-03

**Authors:** I V Kostopoulos, A A Tsakiridou, D Pavlidis, A Megalakaki, S I Papadhimitriou

**Affiliations:** 1 Hematology Laboratory, ‘G. Gennimatas' Athens Regional General Hospital, Athens, Greece; 2 Department of Hematology, ‘Metaxa' Piraeus Cancer Hospital, Piraeus, Greece

Chronic lymphocytic leukemia (CLL) is the most common form of leukemia in Western countries and accounts for 34% of adult leukemias in the United States^1^. Although the etiology of CLL remains unknown, there is increasing evidence for the pathogenetic role of inherited factors.^2^ The familial subtype of the disease is distinguished from sporadic CLL by the presence of at least one affected relative, with the same or a different chronic lymphoproliferation. According to the usual approach, these families have been analyzed to detect linkage, based on the hypothesis of coinheritance of genetic markers. However, due to the small pedigree sizes, these studies have proved inconclusive in determining any specific gene or non-coding region related to the pathophysiology of CLL.^3, 4^ Moreover, several reports on such cases reveal a significant biologic heterogeneity of familial CLL and indicate that there is no consistent pattern of pathogenesis that would suggest a simple mode of transmission. In this report, we present two siblings diagnosed with CLL, with common chromosomal aberrations and an almost identical pattern of clonal evolution, which is unusual among all published familial cases.

The elder brother presented with an isolated lymphocytosis at the age of 60. Flow cytometry showed that almost all peripheral blood lymphocytes were CD19^+^, CD5^+^, CD20^dim^, CD23^dim^, CD38^+^, with kappa light chain restriction and the diagnosis of CLL (Binet stage A) was established. At first, he was followed-up without any treatment, but three years later the disease progressed to Binet stage C and the patient was started with the FCR protocol. The response was satisfactory, but the clinical course was complicated with severe neutropenia and multiple infections. For the next two years he remained off-treatment but continued to experience recurrent herpetic infections. Then, the disease progressed again and proved refractory to all therapeutic agents administered (including the R-CHOP regimen, chlorambucil/prednisolone, alemtuzumab and bendamustine). The patient finally succumbed to multiple organ system failure, 69 months after initial diagnosis.

His younger sister was diagnosed with CLL (Binet stage B) at the age of 54. She reported a history of thyroid carcinoma treated with radioactive iodine. Flow cytometry analysis of peripheral blood showed infiltration from CD19^+^, CD5^+^, CD20^+^, CD23^+^, CD38^+^ lymphocytes with lambda light chain restriction. She remained off treatment for four years. Then, due to disease progression, she was treated with the FCR protocol which was well tolerated and resulted in a satisfactory response. For the next three years she has been clinically and hematologically stable. She is currently alive and well, having completed a total follow-up period of 88 months.

In both siblings, initial bone marrow karyotype was normal and the IGVH sequence was found unmutated. Interphase FISH study was performed on peripheral blood samples upon diagnosis. Tests for t(11;14)(q13;q32), +12, p53 deletion and IGH rearrangement were negative. The results of the tests for ATM and 13q14 deletion are shown in detail on Table 1. Briefly, both patients were found positive for the two aberrations. However, the count of positive cells in conjuction with data from the cytometry analysis on the same samples indicated that the loss of ATM was present in nearly all B cells, while 13q14 deletion involved a subset of B cells only. Moreover, in the male patient, the test for 13q14 revealed a mosaic of cells with hemizygous and biallelic deletion, while his sister showed hemizygous deletion only. Unfortunately, it was not possible to repeat these assays for the male patient until his death, but every effort will be made to repeat them in his sister in the course of the next reevaluation.

The two siblings presented here were found with common cytogenetic aberrations and a similar pattern of clonal evolution upon presentation, namely 13q14 deletion in a subset of cells characterized by ATM loss. These findings may reflect a common series of oncogenic events contributing to the development and progression of the disease.

Deletion of the 13q14 region is the most common cytogenetic aberration in both sporadic (accounting for up to 50% of the cases) and familial CLL, and is considered a marker of favorable prognosis when seen as the sole abnormality. Bi-allelic or concomitant monoallelic/biallelic deletions are detected in up to 1/3 of sporadic cases with 13q14 loss and have also been reported in the setting of familial occurence.^5, 6^ High density mapping of familial cases has revealed a commonly shared haplotype in a locus near 13q14 that may comprise susceptibility genes for the pathogenesis of CLL. Interestingly, 85% of familial members sharing this region have deletions in 13q14.^7, 8^

11q22 deletions involving the ATM gene, are detected less frequently in CLL and have been correlated with advanced disease stage and aggressive clinical behavior. The ATM gene is believed to have a major role in DNA-damage repair procedures, probably responding to a subset of DNA double strand breaks.^9^ Hence, deletion of the ATM gene results in genomic instability, which may be manifested by the acquisition of various additional aberrations, including deletions in vulnerable regions, such as the 13q14 locus. In support of this notion are data from several previous studies on the cytogenetic profile of CLL, providing details about the presence of common chromosomal aberrations (ie +12, 11q-, 17p-, 13q-) and their combinations.^10, 11, 12, 13, 14, 15^ The relevant results are summarized on Table 2, including our data from the study of 542 CLL patients (unpublished observations). It can clearly be seen that in all series, the number of cases with 11q- as a single abnormality is lower than that of 11q- found together with 13q-, and that the number of cases with 11q-/13q- is much higher than the figure expected, based on the random concurrence of two independent events. Moreover, 11q-/13q- proves to be the most common of all possible combinations of single aberrations. In most previous studies, the authors do not provide details about the rates of each aberration in patients with the 11q-/13q- combination, therefore the discrimination between the primary and secondary event is not possible. However, both in Reddy's and our series, among patients with concurrent 11q- and 13q- and a significant difference in the rates of each finding, there were slightly more cases with the 11q- rate exceeding that for 13q-, than the number of cases with the reverse inequality. Finally, 13q- has been detected as a late-appearing aberration in cases with 11q- detected at presentation.^11^ Therefore, the transition from 11q- sole to the 11q-/13q- combination is a rather usual path of clonal evolution in CLL, evidenced either on diagnosis or during follow-up.

Deletion of ATM has been detected in familial CLL, though not necessarily in all affected relatives. Coexistence with 13q- has also been documented in a few cases. However, to the best of our knowledge, the presence of this combination in two affected siblings with a clear distinction between the primary and secondary event has not been previously reported. Despite its apparent rarity, such an observation may not be a matter of chance. Instead it may represent an unidentified mode of inheritance of susceptibility to the development of the disease, linked to the loss of ATM gene. The resulting genomic instability may lead to the acquisition of additional aberrations, with 13q- being a usual candidate, as in similar sporadic cases. Further studies on the cytogenetic background of familial CLL and additional detailed reports of cases of affected relatives will help to support or disclose the validity of these assumptions.

## Figures and Tables

**Table 1:** Results of the i-FISH tests for ATM and 13q14 deletion

*Patient*	*% Clonal B cells (Flow cytometry)*	*ATM deletion (% positive cells)*	*13q14 deletion (% positive cells)*
#1 (♂)	76	74	13q14x1: 31 13q14x2: 14
#2 (♀)	86	83	13q14x1: 42

All percentages refer to the total WBC of the sample.

**Table 2:** Summary of data from previous studies with regard to double combinations of chromosomal aberrations in sporadic CLL

*Single abnormality*	*Our series (*N=*542)*		*Reddy (*N=*509)*		*Shanafelt et al. (*N=*159)*		*Dewald et al. (*N=*113)*		*Dicker et al. (*N=*132)*		*Haferlach et al. (*N=*500)*		*Berkova et al. (*N=*146)*	
del(13q14)	113		122		36		31		57		215		41	
+12	79		71		17		13		10		38		6	
del(11q22)	15		17		2		3		7		23		2	
del(17p13)	12		12		3		0		2		8		6	
														
*Double combinations*	E	O	E	O	E	O	E	O	E	O	E	O	E	O
del(11q22)/del(13q14)	3	16^a^	4	27^b^	1	8	1	6	3	13	10	35	1	15
del(13q14)/+12	16	8	17	13	4	4	4	4	5	2	17	20	2	6
del(13q14)/del(17p13)	3	10	3	16	1	1	0	2	1	3	4	20	2	16
+12/del(17p13)	2	5	2	2	1	0	0	2	1	0	1	6	1	3
del(11q22)/+12	2	2	3	3	1	0	1	0	1	1	2	3	1	1
del(11q22)/del(17p13)	1	1	1	1	1	0	0	0	1	0	1	5	1	0

Abbreviation: CLL, chronic lymphocytic leukemia; *E*, number of cases expected (rounded up to the next integer) based on the random concurrence of independent events; *N*, total number of cases studied; *O*, actual number of cases.

a %11q-=%13q- (10); %11q->%13q- (4); %11q-<%13q- (2).

b %11q-=%13q- (12); %11q->%13q- (9); %11q-<%13q- (6).
